# Cellular and humoral responses after second and third SARS-CoV-2 vaccinations in patients with autoimmune diseases treated with rituximab: specific T cell immunity remains longer and plays a protective role against SARS-CoV-2 reinfections

**DOI:** 10.3389/fimmu.2023.1146841

**Published:** 2023-04-27

**Authors:** Natalia Egri, Hugo Calderón, Robert Martinez, Mario Vazquez, Verónica Gómez-Caverzaschi, Mariona Pascal, Olga Araújo, Manel Juan, Europa Azucena González-Navarro, José Hernández-Rodríguez

**Affiliations:** ^1^ Department of Immunology, Centre de Diagnòstic Biomèdic, Hospital Clínic of Barcelona, Institut d’Investigacions Biomèdiques August Pi i Sunyer (IDIBAPS), University of Barcelona, Center of the European Reference Network (ERN) for Rare Immunodeficiency, Autoinflammatory and Autoimmune Diseases (RITA) and ERN on Connective Tissue and Musculoskeletal Diseases (ReCONNET); Spanish Center of the Centros, Servicios y Unidades de Referencia (CSUR) and Catalan Center of the Xarxa d’Unitats d’Expertesa Clínica (XUEC) for Autoinflammatory Diseases, Autoimmune Diseases and Primary Immunodeficiencies, Barcelona, Spain; ^2^ Clinical Unit of Autoinflammatory Diseases and Vasculitis Research Unit, Department of Autoimmune Diseases, Hospital Clínic of Barcelona, Institut d’Investigacions Biomèdiques August Pi i Sunyer (IDIBAPS), University of Barcelona, Center of the European Reference Network (ERN) for Rare Immunodeficiency, Autoinflammatory and Autoimmune Diseases (RITA) and ERN on Connective Tissue and Musculoskeletal Diseases (ReCONNET), Spanish Center of the Centros, Servicios y Unidades de Referencia (CSUR) and Catalan Center of the Xarxa d’Unitats d’Expertesa Clínica (XUEC) for Autoinflammatory Diseases, Autoimmune Diseases and Primary Immunodeficiencies, Barcelona, Spain

**Keywords:** COVID - 19, SARS-CoV-2 vaccines, humoral response, cellular response, rituximab, anti CD-20, autoimmune diseases, vasculitis

## Abstract

**Background:**

Humoral and cellular immune responses are known to be crucial for patients to recover from COVID-19 and to protect them against SARS-CoV-2 reinfection once infected or vaccinated.

**Objectives:**

This study aimed to investigate humoral and T cell responses to SARS-CoV-2 vaccination in patients with autoimmune diseases after the second and third vaccine doses while on rituximab and their potential protective role against reinfection.

**Methods:**

Ten COVID-19-naïve patients were included. Three time points were used for monitoring cellular and humoral responses: pre-vaccine to exclude virus exposure (time point 1) and post-second and post-third vaccine (time points 2 and 3). Specific IgG antibodies were monitored by Luminex and T cells against SARS-CoV-2 spike-protein by ELISpot and CoVITEST. All episodes of symptomatic COVID-19 were recorded.

**Results:**

Nine patients with antineutrophil cytoplasmic antibody (ANCA)-associated vasculitis and one with an undifferentiated autoimmune disease were included. Nine patients received mRNA vaccines. The last rituximab infusion was administered for a mean (SD) of 15 (10) weeks before the first vaccine and six patients were CD19-B cell-depleted. After a mean (SD) of 19 (10) and 16 (2) days from the second and third vaccine dose, IgG anti-SARS-CoV-2 antibodies were detected in six (60%) and eight (80%) patients, respectively. All patients developed specific T cell responses by ELISpot and CoVITEST in time points 2 and 3. Previous B cell depletion correlated with anti-SARS-CoV-2 IgG levels. Nine (90%) patients developed mild COVID-19 after a median of 7 months of the third dose.

**Conclusion:**

Rituximab in patients with autoimmune diseases reduces humoral responses but does not avoid the development of T cell responses to SARS-CoV-2 vaccination, which remain present after a booster dose. A steady cellular immunity appears to be protective against subsequent reinfections.

## Introduction

Since the initial cases of Coronavirus Disease-19 (COVID-19) reported in December 2019 in Wuhan (China) to March 2023, approximately 676 million people have been recognized to be infected with severe acute respiratory syndrome coronavirus 2 (SARS-CoV-2) worldwide, and more than 6.8 million people have died (1). The advent of SARS-CoV-2 vaccines by the end of 2020 clearly saved humankind from massive devastation because COVID-19 clinical impact has been minimized and the global mortality rate has drastically reduced since then (2).

Humoral and cellular immune responses are known to be crucial for patients to recover from COVID-19 and to protect them against SARS-CoV-2 reinfection once infected or vaccinated (2). During the entire COVID-19 pandemic, SARS-CoV-2 immune protection has been broadly evaluated by detecting antibodies against the virus with automated standardized methods. Cellular response assessment has not been used on a routine basis because of the complexity, time consumption, and cost over most of the widely-used techniques currently exploring cellular immunity, such as the enzyme-linked immunospot assay (ELISpot) and the enzyme-linked immunosorbent assay (ELISA) QuantiFERON^®^ (an Interferon-Gamma [IFN-γ] Release Assays [IGRAs]) for SARS-CoV-2 (3). In this sense, COVID-19 anti-Viral Immunity based on T cells for Evaluation in a Simple Test (CoVITEST) has been recently described as faster and less expensive than ELISpot and is a reliable method to measure and monitor anti-SARS-CoV-2 specific T cells since it is performed with whole blood instead of the peripheral blood mononuclear cells (PBMC) used in ELISpot (3).

Apart from immunoglobulin (Ig) G and IgM antibodies against SARS-CoV-2, T lymphocytes also play a crucial role in protecting the vaccinated population and convalescent patients against severe forms of COVID-19 infection and re-infection, respectively (2). While levels of vaccine-induced antibodies depend on age and are known to decline at 6 months after SARS-CoV-2 vaccination, an early (before antibody response formation) robust T cell immunity has been observed for up to 1 year after infection (4, 5) and at least 6 months after vaccination (6, 7) and remains irrespective of the age and other risk factors of immune dysfunction (6).

However, a proportion of patients with immune-debilitating diseases have been described to lack complete humoral and/or cellular responses to SARS-CoV-2 vaccination, mainly to messenger RNA (mRNA) vaccines. These conditions include primary immunodeficiencies (8–10), neoplastic diseases (9, 11–13), HIV infection and low CD4 lymphocyte counts (14), and liver cirrhosis (15), as well as patients on hemodialysis (16, 17), recipients of solid organ transplants (9, 16, 18–20), stem cell transplantation (21–24), or CAR T-cell therapy (22, 24, 25), and patients with autoimmune diseases, such as rheumatoid arthritis (RA), spondyloarthropathies, systemic lupus erythematosus (SLE), mixed connective tissue disease, multiple sclerosis, autoimmune hepatitis, and different types of vasculitis, which are in turn treated with a wide variety of immunosuppressive agents (9, 26–32).

With regard to immune responses against SARS-CoV-2 mRNA vaccines in patients with autoimmune diseases treated with rituximab and other CD20-depleting agents, overall results reported inconsistent humoral responses (ranging from 9.1% to 68.4%) but stronger specific T cell responses (ranging from 64% to 100% of vaccinated patients) (30, 31, 33–40).

The present study was designed to investigate humoral and T-cell responses to SARS-CoV-2 vaccination in rituximab-treated patients with autoimmune diseases without a previous SARS-CoV-2 infection after the second vaccine dose. The contribution of a third (booster) vaccine dose in humoral and cellular responses was subsequently evaluated. In addition, measurements of T-cell responses by ELISpot were compared to those obtained with CoVITEST.

## Patients and methods

### Patients

Ten COVID-19-naïve patients with systemic autoimmune diseases treated with rituximab at the Department of Autoimmune Diseases, Hospital Clinic of Barcelona, were consecutively included between May 2021 and January 2022. The absence of SARS-CoV-2 exposure was self-reported by all patients and confirmed by being double negative for humoral and cellular response against SARS-CoV-2.

**Figure 1:**
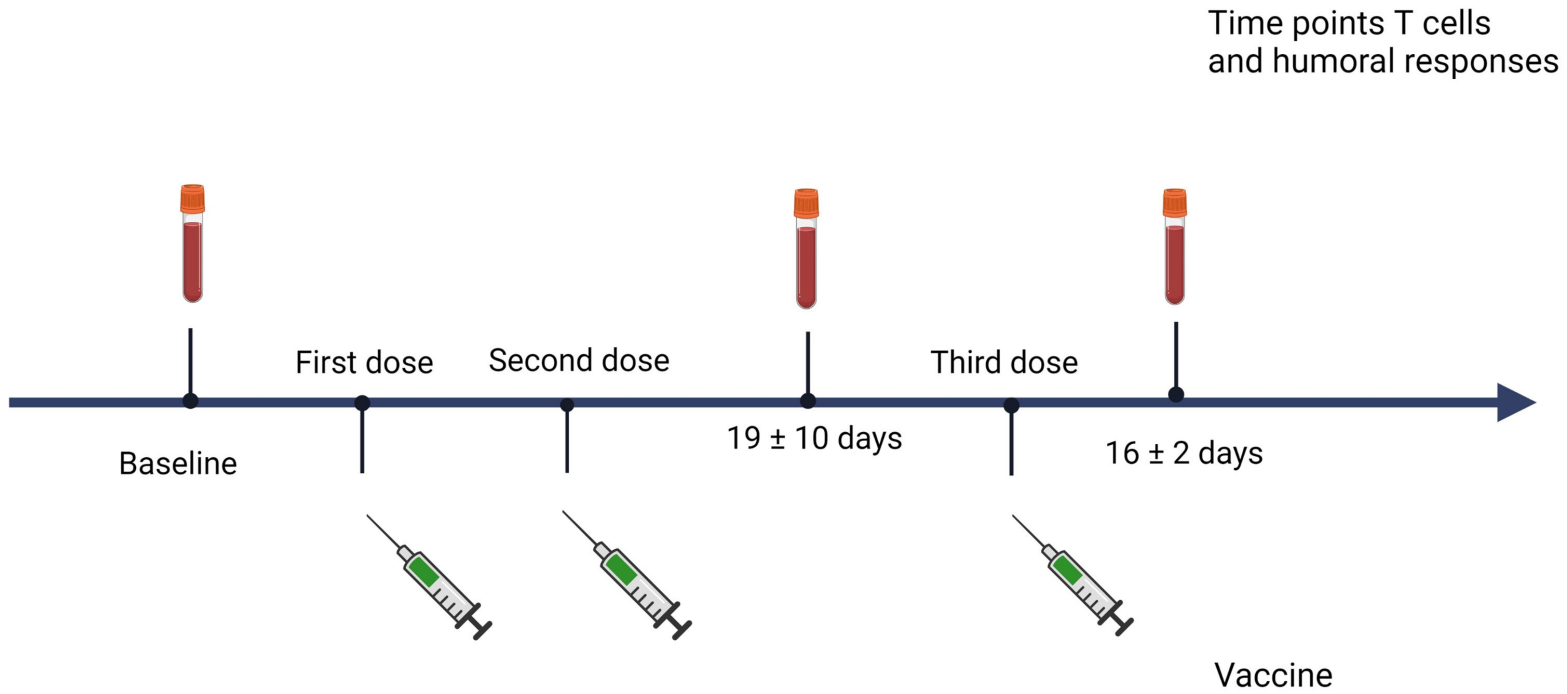
Time of sample collection. Created with BioRender.com.

Patients were monitored for humoral and cellular immunization after vaccination with either mRNA-1273™ (Moderna**®**), BNT162b2™ (Pfizer-BioNTech**®**) mRNA vaccines, or ChAdOx1 nCoV-19™ (Oxford-Astrazeneca**®**) recombinant vaccine. Blood samples were collected at three time points: pre-vaccine or baseline (to exclude virus exposure, time point 1), post-second vaccine (time point 2), and post-third dose vaccination (time point 3) (**Figure 1**). Apart from the quantification of antibodies and specific T cells against SARS-CoV-2, other biological parameters collected included the dose and time of last rituximab infusion, time of vaccines administration, total lymphocyte, CD19+, CD4+, CD8+ counts, and CD4/CD8 cells ratio previous to vaccination. Patients were followed up after the three vaccine doses and all the episodes of symptomatic COVID-19 microbiologically confirmed by real-time quantitative polymerase chain reaction (RT-PCR) or rapid antigen test (RAT) were recorded.

This study was approved by the Research Ethics Committee of the Hospital Clínic of Barcelona (HCB/2020/0967). All procedures were performed in accordance with the ethical principles expressed in the 2013 Declaration of Helsinki. All participants signed informed consent.

### Quantification of IgG antibodies to SARS-CoV-2 by Luminex®

IgG titers were measured by an in-house serological assay based on the Luminex technique that has the benefit of a higher dynamic range than other assays. Detected antibodies were directed against the Receptor-Binding Domain (RBD) of the spike protein of SARS-CoV-2 by Luminex (41). Crude median fluorescent intensities (MFI) were exported using the xPONENT software. The assay cut-off was based on the mean plus three standard deviations of MFIs from 47 negative controls. The data used for the calculations were the ratio of the MFI of the particular individual with the MFI obtained from the donor pool, and a value ≥1 was considered positive. Detection of antibodies from samples of participants previously diagnosed with COVID-19 and disease duration longer than 10 days since the onset of symptoms provided an assay sensitivity of 97% and a specificity of 100% for SARS-CoV-2 IgG antibodies (41).

### T cell responses measurement by IFN-γ ELISpot

To determine the presence of T cell responses against SARS-CoV-2 at different time points, PBMC at a concentration of 2x10^5^ was stimulated in X-VIVO™ 15 medium (Lonza) with PepTivator^®^ SARS-CoV-2 Prot_S (1 µg/mL, Miltenyi Biotec) covering the immunodominant sequence domains of the spike (S) glycoprotein of SARS-CoV-2 (*GenBank MN908947.3 and Protein QHD43423.2)* and nucleocapsid (N) peptide pools. Negative control wells lacked peptides, while positive control wells included anti-CD3-2 mAb. Cells were incubated for 16 to 20 hours at 37°C 5% CO_2_ in pre-coated anti-IFN-γ MSIP white plates (mAb 1-D1K, Mabtech). After incubation, plates were washed five times with PBS (Sigma-Aldrich) and incubated for 2 hours at room temperature with horseradish peroxidase (HRP)-conjugated anti-IFN-γ detection antibody (1 μg/mL; clone mAb-7B6-1; Mabtech). After five further washes with PBS, tetramethylbenzidine (TMB) substrate was added and spots were counted using an automated ELISpot Reader System (Autoimmun Diagnostika GmbH).

In order to quantify positive peptide-specific responses, spots of the unstimulated wells were subtracted from the peptide-stimulated wells, and the results were expressed as Spot forming units SFU/2x10^5^ PBMC. The detection limit of the ELISpot assay was 1/200,000 cells. We determined SARS-CoV-2–specific spots by spot increment defined as stimulated spot numbers ≥ 6 SFU/2x10^5^ PBMC. Only SARS-CoV2 S protein was evaluated by ELISpot in the present study since it is the only useful protein to monitor cellular response in mRNA-vaccinated patients (42).

### T-cell responses evaluation by CoVITEST

Measurements at different points of SARS-CoV-2 specific T lymphocyte responses were also determined by CoVITEST, an in-house method based on the identification of specific T cells immunized against SARS-CoV-2 S and N proteins from whole blood, as previously described (3). Results from the evaluated cases were compared with those obtained by IFN-γ ELISpot.

### Statistical analysis

Descriptive, both continuous and ordinal, variables were presented as means and standard deviations (SD) or medians and interquartile ranges (percentiles 25th to 75th) [IQR 25–75], as appropriate. Differences in means or medians between groups were calculated by Student’s t-test or Mann–Whitney U test. Differences in the ELISpot and CoVITEST values between groups were analyzed by ANOVA, and no post-test corrections were used since multiple comparisons were not needed. Correlations were calculated by the Spearman rank correlation coefficient. Statistical analyses were performed using GraphPad Prism (version 8; GraphPad Software Inc., San Diego, CA, USA). Statistical significance was set at a p-value < 0.05.

## Results

### Baseline characteristics

Nine of the 10 COVID-19-naïve patients (six women and four men) treated with rituximab had been diagnosed with antineutrophil cytoplasmic antibody (ANCA)-associated vasculitis (six with granulomatosis with polyangiitis [GPA] and three with microscopic polyangiitis [MPA]) and one patient had an undifferentiated autoimmune disease. Patients’ mean (SD) age was 55 (17) years. The mean (SD) disease duration was 10 (9) years. Patients were on rituximab during a mean (SD) of 35 (29) months. None received additional immunosuppressive agents other than prednisone, which was taken by four patients at a median [IQR 25-75] dose of 0 [0-2.5] mg/day.

Seven, two, and one patient received the first two doses of mRNA-1273, BNT162b2, and ChAdOx1 nCoV-19 vaccine, respectively. The mean (SD) time between the second and third vaccines was 15 (4) weeks. The same vaccine type was used in all patients except in the patient initially receiving the ChAdOx1 nCoV-19 vaccine, to whom a second BNT162b2 dose was administered.

The CD4+/CD8+ ratio was increased in eight (80%) patients. Previously to the initial vaccination, the last rituximab infusion was administered for a mean (SD) of 15 (10) weeks. Six patients were CD19 B cell-depleted, and four patients had detectable circulating CD19-positive B cells, with a mean (SD) of 5.9 (12.4) cells/mm^3^. Patients’ clinical and immunological characteristics are illustrated in **Table 1**.

**Table 1:** Clinical characteristics, lymphocyte cell populations, and humoral and cellular responses to SARS-CoV-2 vaccines of patients with autoimmune diseases treated with rituximab in the present study.

	Patient 1	Patient 2	Patient 3	Patient 4	Patient 5	Patient 6	Patient 7	Patient 8	Patient 9	Patient 10	Overall
Patients’ general characteristics											
** Autoimmune disease**	GPA	GPA	GPA	GPA	GPA	GPA	MPA	MPA	MPA	IAD	6 GPA/4 MPA/1 UAD
** Sex**	F	F	F	M	M	M	F	F	M	F	6 F/4 M
** Current age (years)**	42	20	68	59	52	44	73	76	64	52	55 ± 17
** Time from disease onset (years)**	18	3	12	2	31	11	1	2	8	10	10 ± 9
** Time on rituximab (months)**	33	30	15	13	66	100	6	24	22	45	35 ± 29
** Prednisone dose (mg/day)**	0.0	0.0	7.5	2.5	0.0	0.0	10.0	2.5	0.0	0.0	0 [0-2.5]
** Vaccine types administered**	mRNA-1273	mRNA-1273	ChAdOx1-S*	mRNA-1273	BNT162b2	mRNA-1273	mRNA-1273	mRNA-1273	mRNA-1273	BNT162b2	7 mRNA-1273/ 2 BNT162b2/ 1 ChAdOx1-S
Data previous to the first vaccine dose											
** Time from the last rituximab dose to the first vaccine (weeks)**	26	4	5	10	13	34	14	15	7	26	15 ± 10
** Lymphocytes (cells/mm^3^)**	1300	2000	2300	1600	2100	1900	1500	1100	2500	1400	1770 ± 460
** CD4/CD8 cells (ratio)**	1.6	–	3.1	1.5	0.6	1.5	0.9	3.2	1.8	1.5	1.5 [1.2-1.8]
** CD19-B cells (cells/mm^3^)**	16.9	0	2.3	0	0	38	1.5	0	0	0	0 [0-5.9]
First and second vaccine doses											
** Time from the second vaccine to response evaluation (days)**	19	15	14	15	29	42	15	9	15	15	19 ± 10
** IgG anti-RBD SARS-CoV-2 (MFI ratio)**	7.04	1.66	0.24	2.29	0.38	14.88	7.41	0.12	0.67	2.27	1.97 [0.3-7.1]
** ELISpot T-cell response**	92	44	10	9	35	13	10	64	49	10	24 [10-53]
** CoVITEST T-cell response**	24	55	18	NP	NP	16	NP	NP	NP	19	19 [17-39.5]
Data previous to the third vaccine dose											
** Time from the last rituximab dose to the third vaccine (weeks)**	10	24	12	4	34	12	3	28	23	13	16 ± 10
** Lymphocytes (cells/mm^3^)**	1400	1300	2800	2000	2100	2100	4100	900	3500	1700	2190 ± 1006
** CD4/CD8 cells (ratio)**	1.6	2.2	2.1	2.2	0.6	1.5	0.7	2.5	1.8	2.4	2 [1.3-2.2]
** CD19-B cells (cells/mm^3^)**	0	0	5.6	0	2.8	107	8.2	49.5	0	0	1.4 [0-18.5]
Third vaccine dose											
** Time from the third vaccine to response evaluation (days)**	15	15	15	15	15	15	20	15	15	15	16 ± 2
** IgG anti-RBD SARS-CoV-2 (MFI ratio)**	1.73	1.92	0.36	2.56	0.24	12.30	6.05	10.69	1.35	1.65	1.83 [1.1-7.2]
** ELISpot T-cell response**	22	28	7	6	14	10	9	14	10	23	12 [9-22]
** CoVITEST T-cell response**	16	23	34	NP	NP	68	NP	NP	NP	27	27 [19.5-51]
Patients’ follow-up after the third vaccine dose											
** Follow-up from study initiation (months)**	19	18	19	20	19	18	17	17	17	18	18 ± 1
** Follow-up from the third vaccine (months)**	14	13	14	13	14	11	13	14	11	14	13 ± 1
** COVID-19 (microbiologically confirmed)** **	Yes	Yes	Yes	Yes#	Yes	Yes	Yes	Yes	No#	Yes	Yes
** Time COVID-19 after 3rd vaccine (months)**	2	7	7	8	3	5	7	8	–	6	7 [4-7]

COVID-19, COronaVIrus Disease-19; CoVITEST, Covid19 anti-Viral Immunity based on T cells for Evaluation in a Simple Test; ELISpot, Enzyme-linked immunospot assay; F, Female; GPA, Granulomatosis with polyangiitis; IgG, Immunoglobulin G; M, Male; MFI, Mean fluorescence intensity; MPA, Microscopic polyangiitis; NP, Not performed; RBD, Receptor-Binding Domain; SARS-CoV-2, Severe acute respiratory syndrome coronavirus 2; UAD, Undifferentiated autoimmune disease.

* In patient 3, after the initial ChAdOx1-S vaccine, the third vaccine administered was BNT162b2.

** Seven patients suffered from mild COVID-19 after the third vaccine dose against SARS-CoV-2.

# Five months after the third dose, patient 4 and patient 9 received a fourth vaccine dose of mRNA-1273 and BNT162b2, respectively. Patient 4 presented with mild COVID-19 symptoms 6 months after the fourth vaccine dose.

### Humoral responses after second and third vaccine doses

**Figure 2:**
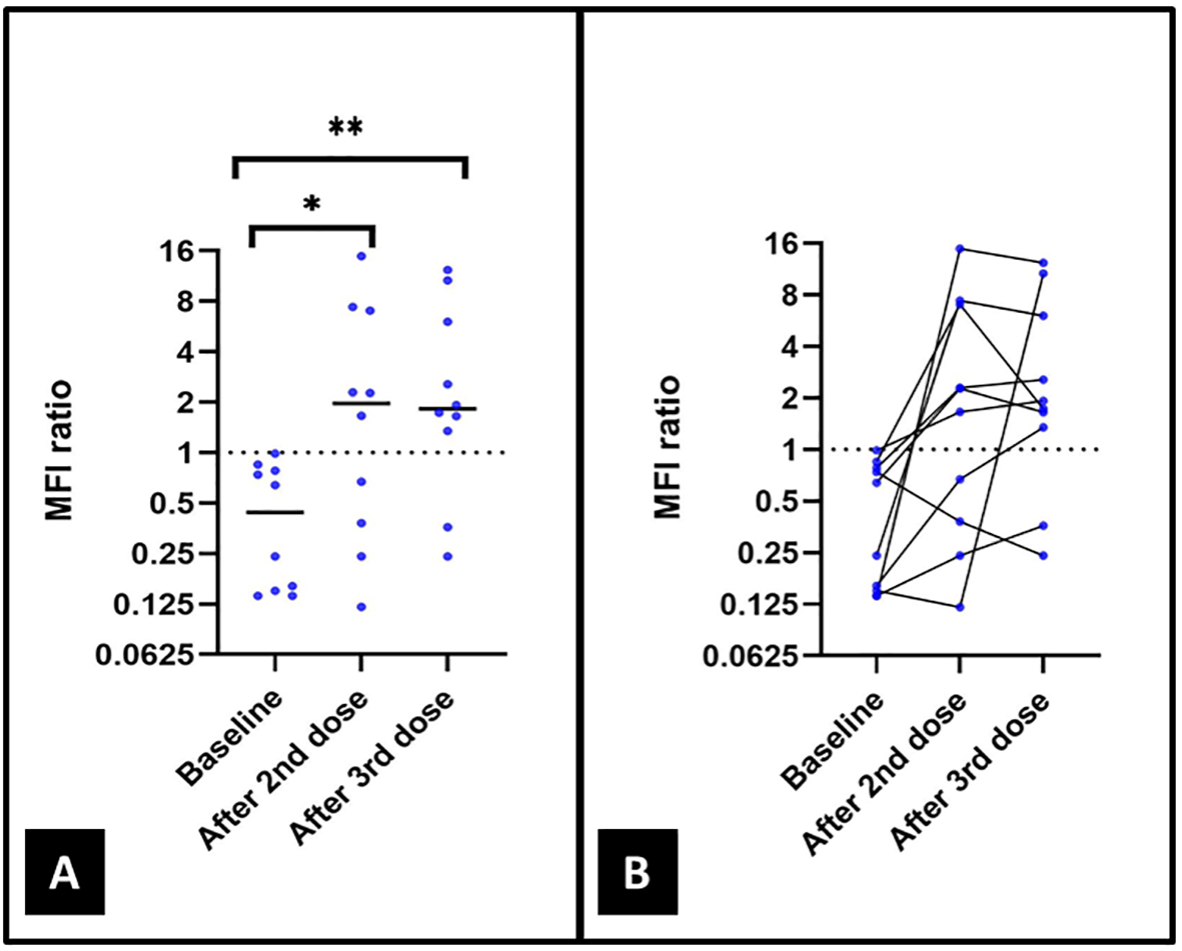
**(A)** Levels of IgG antibodies against the Receptor Binding Domain (RBD) of the spike glycoprotein of SARS-CoV-2 by Luminex in the 10 included patients at baseline and after the second and the third vaccine doses. **(B)** Changes in levels of IgG antibodies against RBD of the spike protein of SARS-CoV-2 in all-time points collected. p value= 0.02*; p value= 0.01**; no statistical significance was found between IgG levels after the second and third vaccinations (p value= 0.72).

After a mean (SD) of 19 (10) days and 16 (2) days following the second and third vaccine dose, respectively, and a mean (SD) period between vaccines of 15 (4) weeks, IgG antibodies were detected after the second immunization in six (60%) patients and eight (80%) patients after the third immunization. Compared to IgG baseline levels, IgG titers significantly increased after the second and third vaccinations (**Figure 2A**). However, no statistically significant differences were observed in median [IQR 25-75] IgG titers after the second (1.97 [0.3-7.1]) and third (1.83 [1.1-7.2]) vaccine doses (p value= 0.72) (**Table 1** and **Figures 2A, B**).

A positive correlation was found between B cell depletion and IgG anti-SARS-CoV-2 levels (Spearman’s correlation coefficient [R_s_]= 0.42 and p value= 0.029). However, among 11 determinations with the absence of B cells, eight (73%) of them had detectable antibodies to SARS-CoV-2.

### Specific T cell responses by ELISpot and CoVITEST after the second and third vaccine doses

**Figure 3:**
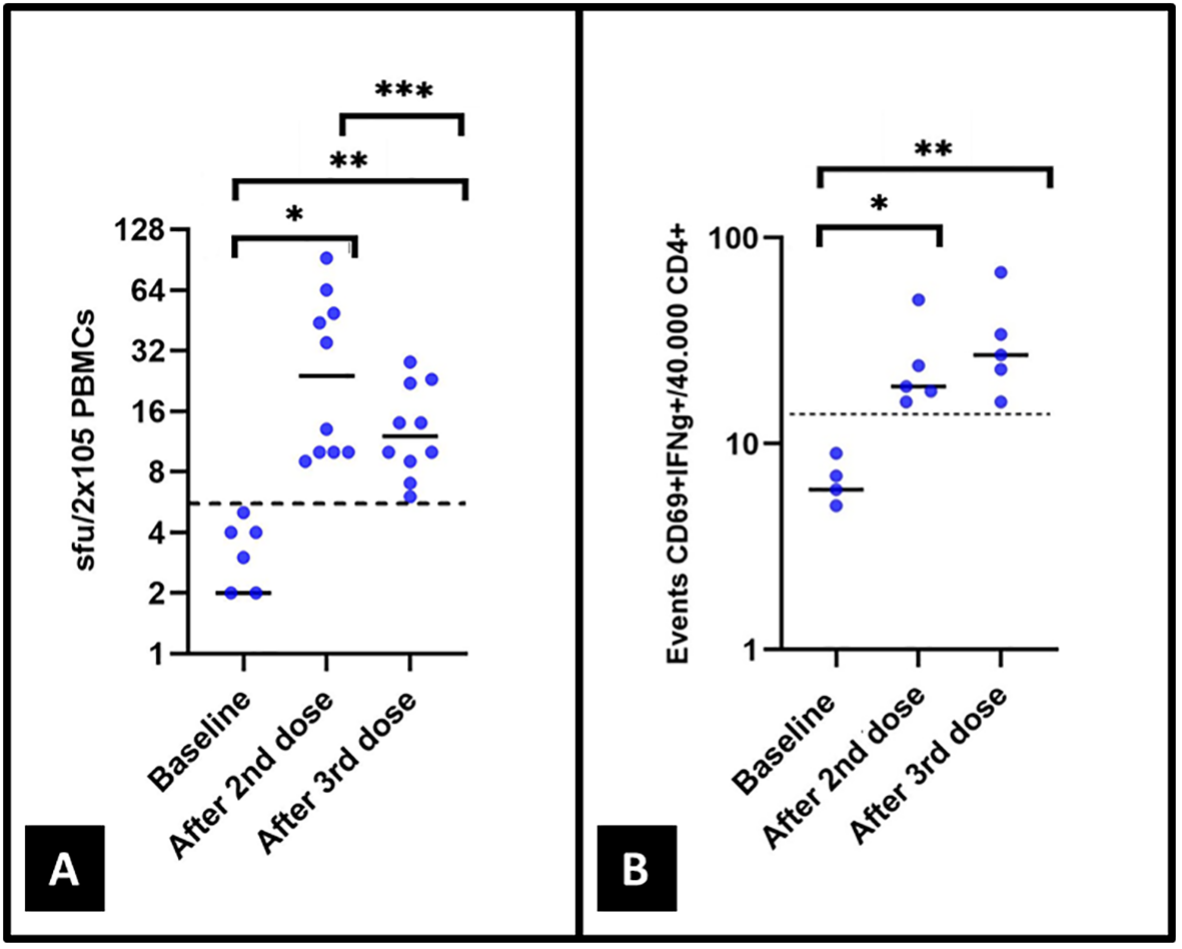
The T-cell response was measured in 10 and 5 patients by **(A)** ELISpot and **(B)** CoVITEST, respectively, both at baseline and after the second and third vaccine doses. In **(A)**, p values of 0.02*, 0.007**, and 0.044***. Four patients with 0 spots at baseline are not included in **(A)**. In **(B)**, p values of 0.042* and 0.043**.

As illustrated in **Table 1**, at the same time points for SARS-CoV-2 antibodies detection, all 10 and five (100%) patients had developed specific T cell responses to SARS-CoV-2 S protein by ELISpot and CoVITEST, respectively, after the second vaccine dose and all of them maintained T cell responses after the third vaccine administration. All T cell determinations by ELISpot and CoVITEST following the second and third vaccinations were significantly increased compared with the baseline levels in all patients (**Figures 3A, B**). Although ELISpot values were all positive after the second and third vaccine doses, median values [IQR 25–75] of specific T cell anti-SARS-CoV-2 were lower after the third vaccine compared to the second immunization (24 [10-53] *vs*. 12 [9-22]; p=0.044) (**Figure 3A**). No statistically significant differences between the extractions after the second and third immunization were observed for CoVITEST (19 [17-39.5] vs. 27 [19.5-51]; p=0.59) (**Figure 3B**).

### SARS-CoV-2 infection during the entire follow-up

During a mean (SD) follow-up period of 13 (1) months since the third vaccine dose, after a median [IQR 25-75] of 7 [4-7] months, nine (90%) patients suffered mild microbiologically proven (by PCR or RAT) COVID-19, clinically manifested with fever or low-grade fever, headache, musculoskeletal, and/or upper respiratory tract symptoms. Only one patient was admitted for a concomitant bacterial respiratory infection due to a chronic lung disease secondary to GPA-related bronchial obstructions and bronchiectasis and treated with bronchodilators and broad-spectrum antibiotics. Patients’ follow-up data is depicted in **Table 1**.

## Discussion

Humoral and cellular-specific responses against SARS-CoV-2 are essential to protect patients from severe forms of COVID-19 (2, 3). While humoral immunity has been widely and routinely used to prove or monitor the immune response to SARS-CoV-2 infection or its vaccination, cellular responses by specific T lymphocytes sensitized to the infection or vaccination have been used mostly for research purposes (3).

Cellular immunity against SARS-CoV-2 is clearly protective from severe forms of the disease since it was demonstrated that pre-existing non-spike memory T cells protection (from cold coronaviruses) conferred cross-reactive protection to secondary exposure in SARS-CoV-2-naïve individuals (43, 44). The protective role of T cells in SARS-CoV-2 infection is also supported by the fact that early induction of IFN-γ producing SARS-CoV-2-specific T cells has been associated with milder diseases and accelerated viral clearance in patients with COVID-19 (45). In addition, T-cell responses have been developed in individuals without detectable humoral response after asymptomatic or mild COVID-19 (46, 47) or vaccination (9), and have been reported to be effective in the recovery of two patients with SARS-CoV-2 infection in the absence of B-cells and directed antibodies due to X-linked agammaglobulinemia (48).

Specific T-cell responses to SARS-CoV-2 infection or vaccination in the general population have been reported to be close to 100%. However, its duration is not clear yet. In this sense, a robust T cell immunity against SARS-CoV-2 has been reported to be maintained in the great majority of patients for up to 9 to 12 months following COVID-19 recovery regardless of the severity of the infection (4, 5), and at least for 6 months after mRNA vaccination (7). Of note, memory T-cell responses to SARS-CoV-1 have been detected 17 years after the outbreak of SARS in 2003, supporting long-lasting protection against COVID-19 once T cells have been sensitized to the coronavirus (49)

The disappearance of circulating IgG SARS-CoV-2 antibodies is part of the normal kinetics of natural immunity since once the infection is under control, the adaptive immune response declines and allows the immune system to be ready to react against new threats. If a robust memory response has been armed, reinfection can be abrogated even before it is fully established (50). The constant finding of new SARS-CoV-2 variants due to numerous mutations in the spike RBD entails that new coronaviruses can potentially escape from humoral immunity. However, these pathogens do not escape from memory T cells induced by previous contact with the wild-type virus or vaccines since specific T cells react against conserved regions within any of the viral proteins of the different SARS-CoV-2 variants. This cellular immunity finally protects the patient from severe diseases and also contributes to reducing transmission (50).

Contrarily to the general (healthy) population, cellular responses to SARS-CoV-2 have been found to be reduced in vulnerable populations, such as patients with primary immunodeficiencies or those secondarily immunocompromised (8–25), including those with autoimmune diseases (9, 26–32). Most immunosuppressive agents, including glucocorticoids, mycophenolate, methotrexate, tumor necrosis factor (TNF) inhibitors, tocilizumab, abatacept, and rituximab have been associated with poor humoral responses in patients with autoimmune diseases after SARS-CoV-2 vaccination (26–28, 32), and the highest risk has been identified with the use of rituximab (26). A retrospective series of 496 immunocompromised patients with debilitating diseases, including 149 with autoimmune diseases, found attenuated humoral and cellular responses after the initial SARS-CoV-2 vaccine since 62% of the cases seroconverted for anti-spike 1 IgG and 71% developed cellular responses. Moreover, the proportion of positive response only increased to 69% and 73% of patients following a booster dose, which represents poor increases of 10% and 3%, respectively. Treatments associated with low humoral response rates after primary vaccination have included the use of anti-CD20 monoclonal antibodies, sphingosine 1-phosphate (S1P) receptor modulators, and mycophenolate. However, only the administration of S1P receptor modulators and mycophenolate, but not rituximab, has been associated with low cellular response rates (9).

Among immunosuppressive drugs, anti-CD20-directed agents selectively and rapidly reduce circulating B cells, preserving tissue B cells and antibody-producing B cells (51). The use of rituximab as the first anti-CD20 monoclonal antibody became an inflection point in the successful control and cure of several B cell-mediated diseases, such as lymphoproliferative disorders and chronic inflammatory and autoimmune diseases, such as ANCA-associated vasculitis. Apart from rituximab, the anti-CD20 drug is still the most frequently used; second- and third-generation anti-CD20 monoclonal antibodies, such as ocrelizumab and ofatumumab, have been developed and also used with good results in different autoimmune diseases (51).

As depicted in **Table 2**, SARS-CoV-2 vaccines in patients with ANCA-associated vasculitis, multiple sclerosis, RA, SLE, inflammatory myopathies, and others, rituximab has been associated with poor humoral responses, ranging from 9.1% to 68.4% positivity after the two first vaccine doses (30, 31, 33–40, 52, 53). However, despite the incomplete humoral response to SARS-CoV-2 mRNA vaccines, patients with multiple sclerosis treated with anti-CD20 agents develop specific cellular responses up to 90 days after vaccination in 64% to 100% of patients (30, 37–40). Similar results, in terms of developing attenuated humoral but full T cell responses, ranging from 72.7% to 100% of patients have also been reported in patients with different systemic autoimmune diseases treated with rituximab (30, 31, 33–36). In patients with chronic lymphocytic leukemia and lymphoma treated with rituximab, a lack of humoral but preserved cellular immunity has been similarly reported (11, 54).

**Table 2:** Studies analyzing humoral and cellular responses to SARS-CoV-2 vaccines in patients with autoimmune diseases treated with anti-CD20 agents.

	N patients	Anti-CD20 agent used	Autoimmune disease	Time from last anti-CD20 dose *(months)*	Vaccine type	Number of vaccine doses	Time from vaccine to response evaluation *(days)*	Humoral response %	Cellular response %
**Fabris et al. (** 33)	11	Rituximab	AAV, MCTD, ASS, DM	3.3 ± 2	BNT162b2	2 doses	21-28	9.1	72.7
**Stefanski et al. (** 34)	19	Rituximab	RA, AAV	9 (IQR 6-13.5)	BNT162b2	2 doses	21-28	68.4	73.7
**Bitoun et al. (** 31)	24	Rituximab	RA, SSj, SLE, Myositis, SSc	5.4 (0–9.8)	BNT162b2	2 doses	28-56	29*	100
**Bonelli et al. (** 35)	5	Rituximab	AAV, myopathy, SLE, MCTD	5 (4-12)	BNT162b2	2 doses	12-23	40	100
**Westhoff et al.** (36)	9	Rituximab	AAV, IgAV, MGN	2.5 (2-7)	BNT162b2	2 doses	21	22.2	86
**Firinu et al. (** 30)	18	Rituximab, ocrelizumab	MS, SLE, RA, MCTD	3.3 (IQR 1.4)	BNT162b2	2-3 doses	21-28	50-55	64-79
**Zabalza et al. (** 37)	175**	Rituximab, ocrelizumab, ofatumumab	MS and other autoimmune diseases**	3 (1.6-4.8)	8 BNT162b2/164 mRNA-1273	2 doses	60 ± 18	45.6	86.4#
**Gadani et al.** (38)	38	Ocrelizumab (n=37), rituximab (n=1)	MS	5.5 ± 3.6	23 BNT162b2/10 mRNA-1273	2 doses	71.5 ± 14	56.4	96.9
**Apostolidis et al. (** 39)	20	Ocrelizumab (n=19), rituximab (n=1)	MS	NS	BNT162b2/mRNA-1273	2 doses	105¦	50	100
**Bajwa et al. (** 40)	54	Ocrelizumab	MS	4 (0.2-11)	BNT162b2	2-3 doses	14-28	33.3	100
**Present series**	10	Rituximab	AAV, UAD	4.5 ± 2.8	3 BNT162b2/ 7 mRNA-1273	2-3 doses	19 ± 10 16 ± 2	60-80	100

AAV, Antineutrophil cytoplasmic antibodies-associated vasculitis; ASS, Antisynthetase syndrome; DM, Dermatomyositis; IgAV, Immunoglobulin A–associated vasculitis; IgG4-RD, IgG4-related disease; MCTD, mixed connective tissue disease; MGN, membranous glomerulonephritis; MS, Multiple sclerosis; NS, Not stated; NT, Not tested; RA, Rheumatoid arthritis; SSc, Systemic sclerosis; SSj, Sjögren’s syndrome; UAD, Undifferentiated autoimmune disease.

All values are provided as median (range or interquartile rate [IQR]) or mean ± standard deviation.

* No humoral response was seen in any patients who received rituximab 6 months prior SARS-CoV-2 vaccine.

** 139 patients with MS and 36 patients with other autoimmune diseases were treated with anti-CD20 agents (rituximab, ocrelizumab, or ofatumumab). The latter group included neurologic autoimmune disorders (other than MS), IgG-4-related disease, and different types of vasculitis and nephropathies.

# Overall cellular response in patients on anti-CD20 agents was 86.4%, but 91.4% of patients without humoral response had a detectable T cellular response.

¦ This value has been estimated from a published figure (39).

Overall, these previous results from patients with autoimmune diseases treated with rituximab were comparable to those found in our patients, in whom humoral and cellular responses were obtained in 60% and 100% of them after the first two SARS-CoV-2 vaccine doses, respectively. Although no statistically significant increase in the median IgG titer was observed, an increase of 25% (from 60% to 80%) in the humoral response from the second to the third vaccine dose was detected. This would appear to be a significant improvement, particularly if extrapolated to a larger cohort. However, both second and third (booster) doses maintained cellular responses in 100% of cases.

In patients treated with rituximab and vaccinated against SARS-CoV-2, B cell depletion has been identified as the main independent factor contributing to the lack of antibody response (9, 55). For this reason, COVID-19 vaccines have been recommended to be administered at least after 6 months after the previous rituximab infusion (56). In our series, B cell counts of previous SARS-CoV-2 vaccination similarly correlated with the presence of specific IgG antibodies. However, in eight of the 11 determinations of patients with complete B cell depletion, IgG anti-SARS-CoV-2 antibodies were detected. Therefore, the absence of B cells does not seem to totally preclude the capacity to produce specific anti-SARS-CoV-2 antibodies. In addition, after following vaccinated patients for a mean of 13 months from the third dose, the protective effect against severe reinfection was observed during at least a median of 7 months period in which 90% of our rituximab-treated patients suffered mild COVID-19. Therefore, vaccine protection after the third dose occurred without substantial immunological changes between the second and the third vaccine doses.

With regard to the studies evaluating vaccine boosters or revaccination in the general population, an increase in levels of IgG antibodies and specific T cells against SARS-CoV-2 has been observed after every vaccine dose (57, 58). However, other studies found that the frequency and intensity of T cell responses have not been significantly boosted by a repeated vaccination (59). In addition, in the studies showing a good response to revaccination, humoral and cellular response after initial vaccination (and previous to vaccine booster administration) seemed to be already protective in most patients (57, 58). This fact shows that the real need or the right time for revaccination remains to be elucidated. Moreover, the main clinical trials about COVID-19 vaccines have not included immunocompromised individuals to prove the real effect in this vulnerable population in whom rapid loss or no humoral response to SARS-CoV-2 infection or vaccination is expected. By contrast, this group of patients has been selected for routine revaccinations (57, 58). The onset and exacerbation of different autoimmune diseases have been increasingly associated with COVID-19 vaccination or revaccination, mostly those vaccinated with mRNA vaccines (60–66). In this sense, the relapse rate in a series of 5,121 patients with autoimmune diseases after SARS-CoV-2 vaccination has been reported in 4.4%, with 0.6% of patients suffering severe flares (67).

The present study has several limitations, including the reduced number of patients with autoimmune diseases included and the relatively short period to prove the duration of specific cellular responses in these patients. No healthy controls (or patients with autoimmune diseases not receiving rituximab) were planned to be included as a comparison group for better characterizing humoral and cellular responses. However, the strengths of the study are based on the homogeneity of patients and the time-point sampling. In addition, these results emphasize the value of ELISpot and CoVITEST as two accurate methods to detect initial cellular responses after COVID-19 or in SARS-CoV-2 vaccinated immunocompromised individuals. This study also provides information about the clinical response to vaccines anti-SARS-CoV-2 in real life, highlighting the value of a maintained T cell response in developing mild forms of COVID-19 after new exposures and protection against severe disease.

## Conclusions

We identified a robust T cell response in patients with autoimmune diseases treated with rituximab despite a reduced humoral response to SARS-CoV-2 vaccination. A booster (third) vaccine dose slightly increases SARS-CoV-2 antibody levels in some patients and maintains cellular responses in all patients.

Our findings, which are similar to those found in previous studies using anti-CD20 agents in patients with autoimmune diseases vaccinated against SARS-CoV-2, should have implications for clinical decision-making and public health policies in the care of immunosuppressed patients and other vulnerable populations. Hopefully, further revaccination surveillance studies will soon clarify the real protective duration of cellular responses to SARS-CoV-2 exposure and vaccinations, and the potential causal relationship between vaccines and new-onset or relapses of autoimmune diseases. By now, in absence of specific antibodies to SARS-CoV-2 after the first vaccine doses, rapid monitoring of specific cellular responses should be warranted in patients before administering unnecessary booster doses if a cellular response is present.

## Data availability statement

The raw data supporting the conclusions of this article will be made available by the authors, without undue reservation.

## Ethics statement

This study was approved by the Research Ethics Committee of the Hospital Clinic of Barcelona (HCB/2020/0967). The patients provided their written informed consent to participate in this study.

## Author contributions

NE, HC, MP, MJ, EG-N, and JH-R: study design. NE and JH-R: sample and data collection, data analysis, interpretation of the results, and manuscript drafting. VG-C and OA: sample and data collection. NE, RM, and MV: samples processing. All authors contributed to the article and approved the submitted version.

## References

[1] COVID-19 dashboard by the center for systems science and engineering (CSSE) at johns Hopkins university (JHU) (2020). Available at: https://www.arcgis.com/apps/dashboards/bda7594740fd40299423467b48e9ecf6 (Accessed 21 March 2023).

[2] Vardhana S Baldo L Morice WG 2nd Wherry EJ. Understanding T cell responses to COVID-19 is essential for informing public health strategies. Sci Immunol (2022) 7:eabo1303. doi: 10.1126/sciimmunol.abo1303 35324269 PMC10344642

[3] Egri N Olivé V Hernández-Rodríguez J Castro P De Guzman C Heredia L. CoVITEST: a fast and reliable method to monitor anti-SARS-CoV-2 specific T cells from whole blood. Front Immunol (2022) 13:848586. doi: 10.3389/fimmu.2022.848586 35865538 PMC9295597

[4] Feng C Shi J Fan Q Wang Y Huang H Chen F. Protective humoral and cellular immune responses to SARS-CoV-2 persist up to 1 year after recovery. Nat Commun (2021) 12:4984. doi: 10.1038/s41467-021-25312-0 34404803 PMC8370972

[5] Flieder T Fischer B von Bargen K Peter A Knabbe C Birschmann I. Humoral and cellular immune response levels at a 1-year follow-up after mild COVID-19. J Clin Virol (2022) 154:105236. doi: 10.1016/j.jcv.2022.105236 35896052 PMC9262650

[6] Kato H Miyakawa K Ohtake N Yamaoka Y Yajima S Yamazaki E. Vaccine-induced humoral response against SARS-CoV-2 dramatically declined but cellular immunity possibly remained at 6 months post BNT162b2 vaccination. Vaccine (2022) 40:2652–5. doi: 10.1016/j.vaccine.2022.03.057 PMC8960126 35370020

[7] Goel RR Painter MM Apostolidis SA Mathew D Meng W Rosenfeld AM. mRNA vaccines induce durable immune memory to SARS-CoV-2 and variants of concern. Science (2021) 374:abm0829. doi: 10.1126/science.abm0829 34648302 PMC9284784

[8] Ainsua-Enrich E Pedreno-Lopez N Bracke C Avila-Nieto C Rodriguez de la Concepcion ML Pradenas E. Kinetics of immune responses elicited after three mRNA COVID-19 vaccine doses in predominantly antibody-deficient individuals. iScience (2022) 25:105455. doi: 10.1016/j.isci.2022.105455 36320330 PMC9613776

[9] Yang LM Costales C Ramanathan M Bulterys PL Murugesan K Schroers-Martin J. Cellular and humoral immune response to SARS-CoV-2 vaccination and booster dose in immunosuppressed patients: an observational cohort study. J Clin Virol (2022) 153:105217. doi: 10.1016/j.jcv.2022.105217 35714462 PMC9188451

[10] Amodio D Ruggiero A Sgrulletti M Pighi C Cotugno N Medri C. Humoral and cellular response following vaccination with the BNT162b2 mRNA COVID-19 vaccine in patients affected by primary immunodeficiencies. Front Immunol (2021) 12:727850. doi: 10.3389/fimmu.2021.727850 34671350 PMC8521226

[11] Lyski ZL Kim MS Xthona Lee D Raue HP Raghunathan V Griffin J. Cellular and humoral immune response to mRNA COVID-19 vaccination in subjects with chronic lymphocytic leukemia. Blood Adv (2022) 6:1207–11. doi: 10.1182/bloodadvances.2021006633 PMC8651482 34872103

[12] Benjamini O Gershon R Haim EB Lustig Y Cohen H Doolman R. Cellular and humoral response to the fourth BNT162b2 mRNA COVID-19 vaccine dose in patients with CLL. Eur J Haematol (2023) 110:99–108. doi: 10.1111/ejh.13878 PMC9874468 36208015

[13] Lasagna A Lilleri D Agustoni F Percivalle E Borgetto S Alessio N. Analysis of the humoral and cellular immune response after a full course of BNT162b2 anti-SARS-CoV-2 vaccine in cancer patients treated with PD-1/PD-L1 inhibitors with or without chemotherapy: an update after 6 months of follow-up. ESMO Open (2022) 7:100359. doi: 10.1016/j.esmoop.2021.100359 34973510 PMC8664661

[14] Antinori A Cicalini S Meschi S Bordoni V Lorenzini P Vergori A. Humoral and cellular immune response elicited by mRNA vaccination against severe acute respiratory syndrome coronavirus 2 (SARS-CoV-2) in people living with human immunodeficiency virus receiving antiretroviral therapy based on current CD4 T-lymphocyte count. Clin Infect Dis (2022) 75:e552–63. doi: 10.1093/cid/ciac238 PMC9047161 35366316

[15] Giambra V Piazzolla AV Cocomazzi G Squillante MM De Santis E Totti B. Effectiveness of booster dose of anti SARS-CoV-2 BNT162b2 in cirrhosis: longitudinal evaluation of humoral and cellular response. Vaccines (Basel) (2022) 10:1281. doi: 10.3390/vaccines10081281 36016169 PMC9415026

[16] Swai J Gui M Long M Wei Z Hu Z Liu S. Humoral and cellular immune response to severe acute respiratory syndrome coronavirus-2 vaccination in haemodialysis and kidney transplant patients. Nephrol (Carlton) (2022) 27:7–24. doi: 10.1111/nep.13974 PMC8646800 34510645

[17] Melin J Svensson MK Albinsson B Winqvist O Pauksens K. Humoral and cellular response to SARS-CoV-2 BNT162b2 mRNA vaccine in hemodialysis patients. BMC Immunol (2021) 22:70. doi: 10.1186/s12865-021-00458-0 34666683 PMC8524400

[18] Cucchiari D Egri N Bodro M Herrera S Del Risco-Zevallos J Casals-Urquiza J. Cellular and humoral response after MRNA-1273 SARS-CoV-2 vaccine in kidney transplant recipients. Am J Transplant (2021) 21:2727–39. doi: 10.1111/ajt.16701 PMC8222867 34036720

[19] Herrera S Colmenero J Pascal M Escobedo M Castel MA Sole-Gonzalez E. Cellular and humoral immune response after mRNA-1273 SARS-CoV-2 vaccine in liver and heart transplant recipients. Am J Transplant (2021) 21:3971–9. doi: 10.1111/ajt.16768 PMC9800111 34291552

[20] Harberts A Schaub GM Ruether DF Duengelhoef PM Brehm TT Karsten H. Humoral and cellular immune response after third and fourth SARS-CoV-2 mRNA vaccination in liver transplant recipients. Clin Gastroenterol Hepatol (2022) 20:2558–66.e5. doi: 10.1016/j.cgh.2022.06.028 PMC9287575 35850415

[21] Bergman P Blennow O Hansson L Mielke S Nowak P Chen P. Safety and efficacy of the mRNA BNT162b2 vaccine against SARS-CoV-2 in five groups of immunocompromised patients and healthy controls in a prospective open-label clinical trial. EBioMedicine (2021) 74:103705. doi: 10.1016/j.ebiom.2021.103705 34861491 PMC8629680

[22] Wu X Wang L Shen L He L Tang K. Immune response to vaccination against SARS-CoV-2 in hematopoietic stem cell transplantation and CAR T-cell therapy recipients. J Hematol Oncol (2022) 15:81. doi: 10.1186/s13045-022-01300-9 35710431 PMC9200932

[23] Manjappa S Phi HQ Lee LW Onstad L Gill DB Connelly-Smith L. Humoral and cellular immune response to covid-19 vaccination in patients with chronic graft-versus-host disease on immunosuppression. Transplant Cell Ther (2022) 28:784.e1–784.e9. doi: 10.1016/j.jtct.2022.08.026 PMC9436787 36058550

[24] Tamari R Politikos I Knorr DA Vardhana SA Young JC Marcello LT. Predictors of humoral response to SARS-CoV-2 vaccination after hematopoietic cell transplantation and CAR T-cell therapy. Blood Cancer Discovery (2021) 2:577–85. doi: 10.1158/2643-3230.BCD-21-0142 PMC8580614 34778798

[25] Jarisch A Wiercinska E Huenecke S Bremm M Cappel C Hauler J. Immune responses to SARS-CoV-2 vaccination in young patients with anti-CD19 chimeric antigen receptor T cell-induced b cell aplasia. Transplant Cell Ther (2022) 28:366 e361–366.e367. doi: 10.1016/j.jtct.2022.04.017 PMC9040419 35472554

[26] Krasselt M Wagner U Nguyen P Pietsch C Boldt A Baerwald C. Humoral and cellular response to COVID-19 vaccination in patients with autoimmune inflammatory rheumatic diseases under real-life conditions. Rheumatol (Oxford) (2022) 61:SI180–8. doi: 10.1093/rheumatology/keac089 PMC8903382 35143648

[27] Monti S Fornara C Delvino P Bartoletti A Bergami F Comolli G. Immunosuppressive treatments selectively affect the humoral and cellular response to SARS-CoV-2 in vaccinated patients with vasculitis. Rheumatol (Oxford) (2023) 62:726–34. doi: 10.1093/rheumatology/keac365 PMC9278207 35736379

[28] Dimitroulas T Tychala A Katsimpourlia E Sidiropoulou E Deuteraiou K Papachristou M. Humoral and cellular response to a third booster dose SARS-CoV- 2 vaccination in patients with autoimmune disease: a case series. Scand J Rheumatol (2022) 51:422–4. doi: 10.1080/03009742.2022.2057000 35546492

[29] Hartl J Ruther DF Duengelhoef PM Brehm TT Steinmann S Weltzsch JP. Analysis of the humoral and cellular response after the third COVID-19 vaccination in patients with autoimmune hepatitis. Liver Int (2023) 43:393–400. doi: 10.1111/liv.15368 PMC9349728 35840342

[30] Firinu D Fenu G Sanna G Costanzo GA Perra A Campagna M. Evaluation of humoral and cellular response to third dose of BNT162b2 mRNA COVID-19 vaccine in patients treated with b-cell depleting therapy. J Autoimmun (2022) 131:102848. doi: 10.1016/j.jaut.2022.102848 35714496 PMC9189114

[31] Bitoun S Henry J Desjardins D Vauloup-Fellous C Dib N Belkhir R. Rituximab impairs b cell response but not T cell response to COVID-19 vaccine in autoimmune diseases. Arthritis Rheumatol (2022) 74:927–33. doi: 10.1002/art.42058 PMC9011892 34962357

[32] Gragnani L Visentini M Lorini S La Gualana F Santini SA Cacciapaglia F. COVID-19 vaccine immunogenicity in 16 patients with autoimmune systemic diseases. lack of both humoral and cellular response to booster dose and ongoing disease modifying therapies. J Transl Autoimmun (2022) 5:100164. doi: 10.1016/j.jtauto.2022.100164 36120415 PMC9472465

[33] Fabris M De Marchi G Domenis R Caponnetto F Guella S Dal Secco C. High T-cell response rate after COVID-19 vaccination in belimumab and rituximab recipients. J Autoimmun (2022) 129:102827. doi: 10.1016/j.jaut.2022.102827 35427999 PMC8995326

[34] Stefanski AL Rincon-Arevalo H Schrezenmeier E Karberg K Szelinski F Ritter J. B cell numbers predict humoral and cellular response upon SARS-CoV-2 vaccination among patients treated with rituximab. Arthritis Rheumatol (2022) 74:934–47. doi: 10.1002/art.42060 PMC9011692 34962360

[35] Bonelli MM Mrak D Perkmann T Haslacher H Aletaha D. SARS-CoV-2 vaccination in rituximab-treated patients: evidence for impaired humoral but inducible cellular immune response. Ann Rheum Dis (2021) 80:1355–6. doi: 10.1136/annrheumdis-2021-220408 33958323

[36] Westhoff TH Seibert FS Anft M Blazquez-Navarro A Skrzypczyk S Doevelaar A. Correspondence on 'SARS-CoV-2 vaccination in rituximab-treated patients: evidence for impaired humoral but inducible cellular immune response'. Ann Rheum Dis (2021) 80:e162. doi: 10.1136/annrheumdis-2021-220756 34272253

[37] Zabalza A Arrambide G Otero-Romero S Pappolla A Tagliani P Lopez-Maza S. Is humoral and cellular response to SARS-CoV-2 vaccine modified by DMT in patients with multiple sclerosis and other autoimmune diseases? Mult Scler (2022) 28:1138–45. doi: 10.1177/13524585221089540 35475363

[38] Gadani SP Reyes-Mantilla M Jank L Harris S Douglas M Smith MD. Discordant humoral and T cell immune responses to SARS-CoV-2 vaccination in people with multiple sclerosis on anti-CD20 therapy. EBioMedicine (2021) 73:103636. doi: 10.1016/j.ebiom.2021.103636 34666226 PMC8520057

[39] Apostolidis SA Kakara M Painter MM Goel RR Mathew D Lenzi K. Cellular and humoral immune responses following SARS-CoV-2 mRNA vaccination in patients with multiple sclerosis on anti-CD20 therapy. Nat Med (2021) 27:1990–2001. doi: 10.1038/s41591-021-01507-2 34522051 PMC8604727

[40] Bajwa HM Novak F Nilsson AC Nielsen C Holm DK Ostergaard K. Persistently reduced humoral and sustained cellular immune response from first to third SARS-CoV-2 mRNA vaccination in anti-CD20-treated multiple sclerosis patients. Mult Scler Relat Disord (2022) 60:103729. doi: 10.1016/j.msard.2022.103729 35334278 PMC8898195

[41] Garcia-Basteiro AL Moncunill G Tortajada M Vidal M Guinovart C Jimenez A. Seroprevalence of antibodies against SARS-CoV-2 among health care workers in a large Spanish reference hospital. Nat Commun (2020) 11:3500. doi: 10.1038/s41467-020-17318-x 32641730 PMC7343863

[42] Wakui M Uwamino Y Yatabe Y Nakagawa T Sakai A Kurafuji T. Assessing anti-SARS-CoV-2 cellular immunity in 571 vaccines by using an IFN-gamma release assay. Eur J Immunol (2022) 52:1961–71. doi: 10.1002/eji.202249794 PMC9874394 36250411

[43] Kundu R Narean JS Wang L Fenn J Pillay T Fernandez ND. Cross-reactive memory T cells associate with protection against SARS-CoV-2 infection in COVID-19 contacts. Nat Commun (2022) 13:80. doi: 10.1038/s41467-021-27674-x 35013199 PMC8748880

[44] da Silva Antunes R Pallikkuth S Williams E Dawen Yu E Mateus J Quiambao L. Differential T-cell reactivity to endemic coronaviruses and SARS-CoV-2 in community and health care workers. J Infect Dis (2021) 224:70–80. doi: 10.1093/infdis/jiab176 33822097 PMC8083569

[45] Tan AT Linster M Tan CW Le Bert N Chia WN Kunasegaran K. Early induction of functional SARS-CoV-2-specific T cells associates with rapid viral clearance and mild disease in COVID-19 patients. Cell Rep (2021) 34:108728. doi: 10.1016/j.celrep.2021.108728 33516277 PMC7826084

[46] Sekine T Perez-Potti A Rivera-Ballesteros O Stralin K Gorin JB Olsson A. Robust T cell immunity in convalescent individuals with asymptomatic or mild COVID-19. Cell (2020) 183:158–168.e114. doi: 10.1016/j.cell.2020.08.017 32979941 PMC7427556

[47] Grifoni A Weiskopf D Ramirez SI Mateus J Dan JM Moderbacher CR. Targets of T cell responses to SARS-CoV-2 coronavirus in humans with COVID-19 disease and unexposed individuals. Cell (2020) 181:1489–1501.e1415. doi: 10.1016/j.cell.2020.05.015 32473127 PMC7237901

[48] Soresina A Moratto D Chiarini M Paolillo C Baresi G Foca E. Two X-linked agammaglobulinemia patients develop pneumonia as COVID-19 manifestation but recover. Pediatr Allergy Immunol (2020) 31:565–9. doi: 10.1111/pai.13263 PMC7264678 32319118

[49] Le Bert N Tan AT Kunasegaran K Tham CYL Hafezi M Chia A. SARS-CoV-2-specific T cell immunity in cases of COVID-19 and SARS, and uninfected controls. Nature (2020) 584:457–62. doi: 10.1038/s41586-020-2550-z 32668444

[50] Wahl I Wardemann H. Sterilizing immunity: understanding COVID-19. Immunity (2022) 55:2231–5. doi: 10.1016/j.immuni.2022.10.017 PMC9595357 36309008

[51] Kaegi C Wuest B Crowley C Boyman O. Systematic review of safety and efficacy of second- and third-generation CD20-targeting biologics in treating immune-mediated disorders. Front Immunol (2021) 12:788830. doi: 10.3389/fimmu.2021.788830 35185862 PMC8847774

[52] Spiera R Jinich S Jannat-Khah D. Rituximab, but not other antirheumatic therapies, is associated with impaired serological response to SARS- CoV-2 vaccination in patients with rheumatic diseases. Ann Rheum Dis (2021) 80:1357–9. doi: 10.1136/annrheumdis-2021-220604 33975857

[53] Furer V Eviatar T Zisman D Peleg H Braun-Moscovici Y Balbir-Gurman A. Predictors of immunogenic response to the BNT162b2 mRNA COVID-19 vaccination in patients with autoimmune inflammatory rheumatic diseases treated with rituximab. Vaccines (Basel) (2022) 10:901. doi: 10.3390/vaccines10060901 35746508 PMC9229869

[54] Bacova B Kohutova Z Zubata I Gaherova L Kucera P Heizer T. Cellular and humoral immune response to SARS-CoV-2 mRNA vaccines in patients treated with either ibrutinib or rituximab. Clin Exp Med (2022) 29:1–9. doi: 10.1007/s10238-022-00809-0 PMC8963888 35352210

[55] Avouac J Miceli-Richard C Combier A Steelandt A Fogel O Mariaggi AA. Risk factors of impaired humoral response to COVID-19 vaccination in rituximab-treated patients. Rheumatol (Oxford) (2022) 61:SI163–8. doi: 10.1093/rheumatology/keab815 PMC8689920 34726701

[56] Troldborg A Thomsen MK Bartels LE Andersen JB Vils SR Mistegaard CE. Time since rituximab treatment is essential for developing a humoral response to COVID-19 mRNA vaccines in patients with rheumatic diseases. J Rheumatol (2022) 49:644–9. doi: 10.3899/jrheum.211152 35232803

[57] Munro APS Feng S Janani L Cornelius V Aley PK Babbage G. Safety, immunogenicity, and reactogenicity of BNT162b2 and mRNA-1273 COVID-19 vaccines given as fourth-dose boosters following two doses of ChAdOx1 nCoV-19 or BNT162b2 and a third dose of BNT162b2 (COV-BOOST): a multicentre, blinded, phase 2, randomised trial. Lancet Infect Dis (2022) 22:1131–41. doi: 10.1016/S1473-3099(22)00271-7 PMC9084623 35550261

[58] Munro APS Janani L Cornelius V Aley PK Babbage G Baxter D. Safety and immunogenicity of seven COVID-19 vaccines as a third dose (booster) following two doses of ChAdOx1 nCov-19 or BNT162b2 in the UK (COV-BOOST): a blinded, multicentre, randomised, controlled, phase 2 trial. Lancet (2021) 398:2258–76. doi: 10.1016/S0140-6736(21)02717-3 PMC8639161 34863358

[59] Maringer Y Nelde A Schroeder SM Schuhmacher J Horber S Peter A. Durable spike-specific T-cell responses after different COVID-19 vaccination regimens are not further enhanced by booster vaccination. Sci Immunol (2022) 7:eadd3899. doi: 10.1126/sciimmunol.add3899 36318037 PMC9798886

[60] Carubbi F Alunno A Santilli J Natali L Mancini B Di Gregorio N. Immune-mediated inflammatory diseases after anti-SARS-CoV-2 vaccines: new diagnoses and disease flares. RMD Open (2022) 8:e002460. doi: 10.1136/rmdopen-2022-002460 36282905 PMC9453424

[61] Nistri R Barbuti E Rinaldi V Tufano L Pozzilli V Ianniello A. Case report: multiple sclerosis relapses after vaccination against SARS-CoV2: a series of clinical cases. Front Neurol (2021) 12:765954. doi: 10.3389/fneur.2021.765954 34744992 PMC8569136

[62] Sagy I Zeller L Raviv Y Porges T Bieber A Abu-Shakra M. New-onset systemic lupus erythematosus following BNT162b2 mRNA COVID-19 vaccine: a case series and literature review. Rheumatol Int (2022) 42:2261–6. doi: 10.1007/s00296-022-05203-3 PMC9468534 36098769

[63] Tan SYS Yee AM Sim JJL Lim CC. COVID-19 vaccination in systemic lupus erythematosus: a systematic review for effectiveness, immunogenicity, flares and acceptance. Rheumatol (Oxford) (2022), keac604. doi: 10.1093/rheumatology/keac604 PMC9620290 36271852

[64] Felten R Kawka L Dubois M Ugarte-Gil MF Fuentes-Silva Y Piga M. Tolerance of COVID-19 vaccination in patients with systemic lupus erythematosus: the international VACOLUP study. Lancet Rheumatol (2021) 3:e613–5. doi: 10.1016/S2665-9913(21)00221-6 PMC8294805 34312612

[65] Quintanilla-Bordas C Gascon-Gimenez F Alcala C Paya M Mallada J Silla R. Case report: exacerbation of relapses following mRNA COVID-19 vaccination in multiple sclerosis: a case series. Front Neurol (2022) 13:897275. doi: 10.3389/fneur.2022.897275 35572939 PMC9091902

[66] Fragoso YD Gomes S Goncalves MVM Mendes Junior E Oliveira BES Rocha CF. New relapse of multiple sclerosis and neuromyelitis optica as a potential adverse event of AstraZeneca AZD1222 vaccination for COVID-19. Mult Scler Relat Disord (2022) 57:103321. doi: 10.1016/j.msard.2021.103321 35158439 PMC8511887

[67] Machado PM Lawson-Tovey S Strangfeld A Mateus EF Hyrich KL Gossec L. Safety of vaccination against SARS-CoV-2 in people with rheumatic and musculoskeletal diseases: results from the EULAR coronavirus vaccine (COVAX) physician-reported registry. Ann Rheum Dis (2022) 81:695–709. doi: 10.1136/annrheumdis-2021-221490 34972811

